# Noradrenergic projections regulate the acquisition of classically conditioned eyelid responses in wild-type and are impaired in kreisler mice

**DOI:** 10.1038/s41598-023-38278-4

**Published:** 2023-07-15

**Authors:** Elena Porras-García, Magdalena Mas-Nieto, José María Delgado-García, Eduardo Domínguez-del-Toro

**Affiliations:** Division of Neurosciences, University Pablo de Olavide, Ctra. de Utrera, Km. 1, 41013 Sevilla, Spain

**Keywords:** Learning and memory, Oculomotor system

## Abstract

During embryonic development, heterozygous mutant kreisler mice undergo ectopic expression of the Hoxa3 gene in the rostral hindbrain, affecting the opioid and noradrenergic systems. In this model, we have investigated behavioral and cognitive processes in their adulthood. We confirmed that pontine and locus coeruleus neuronal projections are impaired, by using startle and pain tests and by analyzing immunohistochemical localization of tyrosine hydroxylase. Our results showed that, even if kreisler mice are able to generate eyelid reflex responses, there are differences with wild-types in the first component of the response (R1), modulated by the noradrenergic system. The acquisition of conditioned motor responses is impaired in kreisler mice when using the trace but not the delay paradigm, suggesting a functional impairment in the hippocampus, subsequently confirmed by reduced quantification of alpha2a receptor mRNA expression in this area but not in the cerebellum. Moreover, we demonstrate the involvement of adrenergic projection in eyelid classical conditioning, as clonidine prevents the appearance of eyelid conditioned responses in wild-type mice. In addition, hippocampal motor learning ability was restored in kreisler mice by administration of adrenergic antagonist drugs, and a synergistic effect was observed following simultaneous administration of idazoxan and naloxone.

## Introduction

It is well accepted that during embryonic development the segmentation of the hindbrain into rhombomeres promotes the early organization of neuronal lineages (giving rise to reticular and motoneurons) and that these rhombomeres are the fundamental units in which these functional properties are specified. Among these properties we must consider the synaptic connectivity and migratory processes leading neurons to occupy different axial positions in the developing neural tube. In the adult, on the other hand, this segmented organization is no longer present, as a result of posterior maturation.

Clinical observations suggest that early development is influencing facial movements. This can be seen in Moebius syndrome^1,2^, in which children may have at birth facial and abducens palsies, but during postnatal development, it is possible these palsies are ameliorated by cortical influences on motor control or by the maturation of neuronal networks in the brainstem. In other cases, as in Joubert syndrome [review in^3^], with an inherited character, we find not only episodic breathing or ataxic problems, but also certain psychomotor retardation.

By using mutants affected specifically in brainstem development, many studies have analyzed functional consequences of abnormal segmentation. Some of them showed respiratory deficiencies caused by reconfiguration of reticular pontine neurons^4–7^, sometimes compromising the survival of individuals. In one case, noradrenergic A5 neurons were involved^4^. These studies show that Hox genes are acting as *Master Genes* in the development of neuronal networks, since the inactivation of Hoxa1 results in the generation of supplementary functional neuronal circuits^5^. As many of these mutants died during postnatal days, it is easy to explain the absence of experimental data demonstrating how hindbrain segmentation influences adult motor behaviors.

Treatment with non-teratogenic doses of retinoic acid during the development of brainstem leads to hyperpneic episodic breathing^8^. This is like that appearing in some human pathologies: episodic breathing in newborns before 40 gestational weeks^9–11^, Cheyne-Stokes syndrome in the adult^12^, or Joubert syndrome, in which have been observed episodic breathing and certain psychomotor retardation have been observed^3^.

In this context, although homozygous Kreisler mice have been extensively studied and have multiple disturbances, heterozygous kreisler mice (+/kr) appear especially interesting, as they can survive, showing no observable anatomical signs, and even suffering an unusual respiratory phenotype: a respiratory frequency at birth increased by 50%^6^. The molecular modification associated to this mutation is particularly selective, consisting of an ectopic expression of Hoxa3 in rhombomere 3 (r3) when it is normally expressed in r5^13^. It has been demonstrated that naloxone administration produces an increase of that accelerated frequency^6^, suggesting an imbalance between the opioid system (inhibiting respiratory responses) and the pontine adrenergic system, normally having an important role in neonatal respiratory maturation [reviewed in^14^].

The involvement of the pontine noradrenergic system in cognitive functions has been discussed regarding its functional participation in processes moving from wakefulness, attention, and memory processes, and finishing in more-complex models related to errors, predictions, making of decisions, and so on^15,16^. An interesting proposal, coming from in vivo recordings, establishes that the phasic activity of coerulear neurons resets cortical circuit activity, allowing the elaboration of behavioral responses^16,17^. Also with regard to these coeruleus-cortex projections, the involvement has been reported of the alpha-adrenergic system in some motor responses, such as prepulse inhibition of the startle reflex^18^. Moreover, it has been demonstrated how these responses are affected in the adult by postnatal injection of the antisense of alpha2A-adrenergic receptor in the locus coeruleus^19^. This treatment affects not only startle responses, but also the development of labyrinth tests and social behaviors^20^.

By using heterozygous kreisler mice, presenting characteristic respiratory phenotypes associated to an alteration of the pontine noradrenergic system coming from embryonic disturbances, and being capable of surviving into adulthood, we could seek possible psychomotor anomalies in them. Our experimental approach has been focused on the eyelid classical conditioning technique, an easy way of motor learning, involving a well-known trigemino-facial reflex, and suggests that both opioid and adrenergic systems are affected in kreisler mice and that they normally participate in the acquisition of conditioned responses.

## Materials and methods

### Experimental subjects

We used adult (3–5 months old) wild-type and mutant (kreisler) male mice on a B6CBA background. Wild-type and kreisler mice were obtained from a stable colony located at the Pablo de Olavide Animal House (Seville, Spain). Animals were housed in common cages (n ≤ 10 per cage) into surgery, after which they were housed individually. Mice were kept on a 12:12 h light–dark cycle with constant ambient temperature (21 ± 1 °C) and humidity (50 ± 7%). Food and water were available ad libitum.

### Ethical statement

Pharmacological and behavioral studies were performed in accordance with the guidelines of the European Union Council (2010/276:33-79/EU) and current Spanish regulations (BOE 34:11370-421, 2013) for the use of laboratory animals in chronic experiments. Experiments were also approved by the Ethics Committee for Animal Care and Handling of the Pablo de Olavide University. The present study is performed in accordance with ARRIVE guidelines (https://arriveguidelines.org).

### Genotyping

DNA was extracted from the tail of the mouse as previously described^21^. The genotype at the *kreisler* locus was determined by a polymerase chain reaction assay for 40 cycles using Taq DNA polymerase and the oligonucleotide primers 5′-GAATTCTTACTCTCTCCCCTAAATTC-3′ and 5′-CAGGGAGAGTTGTTAAGGGATCTTGC-3′ using denaturing, annealing, and extension periods of 94 °C for 30 s, 60 °C for 30 s, and 72 °C for 45 s. An aliquot of the amplified material was digested with HindIII and electrophoresed on a 1.5% agarose gel, which was stained with ethidium bromide (0.2 μg/mL) to determine whether the HindIII site (absent from the mutant allele) is present.

### Surgery

Following a previous description^22^, animals were anaesthetized with a mixture of Ketolar (ketamine, 35 mg/kg) and Rompum (xylazine, 2 mg/kg), i.p., and implanted with bipolar stimulating electrodes on the left supraorbital nerve and with bipolar recording electrodes in the ipsilateral orbicularis oculi muscle. Electrodes were made from 50 µm, Teflon-insulated, annealed stainless steel wire (A-M Systems, Carlsborg, WA, USA), with their tips removed of the isolating cover for ≈ 0.5 mm. Electrode tips were bent as a hook to facilitate a stable insertion in the upper eyelid. Wires were connected to a four-pin socket (RS-Amidata, Madrid, Spain). The socket was fixed to the skull with the help of two small screws and dental cement.

### Classical conditioning of eyelid responses

**Figure 1:**
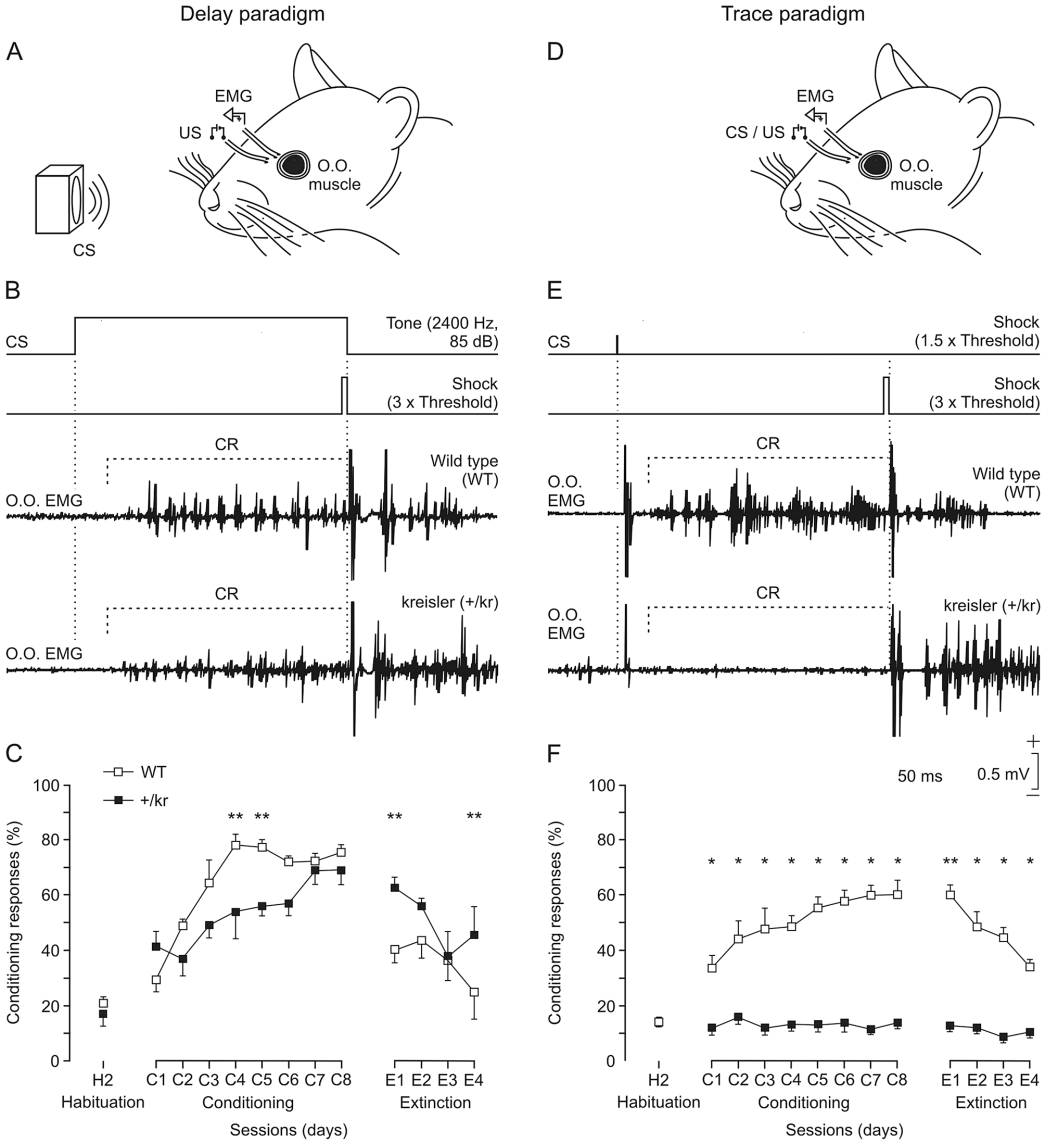
Learning curves for wild-type and kreisler mice. (**A**, **B**, **D**, **E**) Schematic representations of delay (**A**, **B**) and trace (**D**, **E**) conditioning paradigms, illustrating conditioned (CS) and unconditioned (US) stimuli and representative examples of electromyographic (EMG) recordings collected during the eighth conditioning session from wild-type (WT, top) and kreisler (+/kr, bottom) mice. (**C**, **F**) Quantitative analyses of the percentage (± SEM) of conditioned responses (CRs) during the successive sessions for delay (**C**) and trace (**F**) conditioning paradigms. Data collected from the 2nd (H2) habituation session, C1–C8 conditioning sessions, and E1–E4 extinction sessions are illustrated. The percentage of CRs was significantly larger for WT mice for both delay (F_(12,137)_ = 4.912; **, *P* < 0.001; two-way ANOVA and Holm-Sidak method) and trace (F_(12,169)_ = 6.108; *, *P* < 0.05; **, *P* < 0.001; two-way ANOVA and Holm-Sidak method) paradigms. Note that +/kr mice did not present any evidence of CRs during the trace conditioning paradigm.

A week after surgery, the animal was placed in a small (5 × 5 × 10 cm) plastic chamber located inside a Faraday box. Classical conditioning was achieved using delay and trace paradigms (Fig. 1B and E). For the delay paradigm, a 2400 Hz, 85 dB, 250 ms tone was presented as a conditioned stimulus (CS). The unconditioned stimulus (US) consisted of a long (500 µs), strong (3 × threshold), square, cathodal pulse. Threshold was defined as the shock intensity able to evoke a blink in 50% of the cases. Intensities for the US ranged from 0.2 to 0.5 mA. The US co-terminated with the CS. For the trace paradigm, a brief (50 µs), weak (1.5 × threshold), square, cathodal pulse was presented as a CS. The US was the same as that described above and started 250 ms after the end of the CS^22,23^.

Following previous descriptions from our laboratory^22,23^ classical conditioning experiments were organized as follows. Two habituation, eight conditioning, and four extinction sessions were carried out in each animal. A conditioning session consisted of 60 CS–US presentations, and lasted ≈ 30 min. In 10% of the cases, the CS was presented alone. CS–US presentations were separated at random by 30 ± 5 s. For habituation and extinction sessions, only the CS was presented, also for 60 times per session at intervals of 30 ± 5 s. Seven wild-type and six kreisler mice were used for the delay paradigm. For the trace paradigm in controls, eight wild-type and seven kreisler mice were used. In this paradigm, we administrated some drugs as follows: clonidine (five wild-type and nine kreisler mice), idazoxan (seven wild-type and six kreisler mice), naloxone (five wild-type and five kreisler mice), and idazoxan plus naloxone (five wild-type and five kreisler mice).

### Recording procedures

Recording procedures followed previous descriptions from some of us^22,23^. The electromyographic (EMG) activity of the orbicularis oculi muscle was recorded using GRASS P511 differential amplifiers within a bandwidth of 1 Hz to 10 kHz (Grass-Telefactor, West Warwick, RI 02893, USA). The basic properties of blink reflexes were studied in responses evoked by single 50 µs, 2 × threshold, square, cathodal pulses presented at a rate of 1/30 s. Reflex blinks were studied before the initiation of the conditioning sessions.

For criteria, we considered a 'CR' to be the presence of EMG activity during the CS–US period that lasted > 10 ms and was initiated > 30 ms after CS onset. To obtain a quantitative index of CR evolution, the integrated EMG activity recorded during the CS–US interval was averaged and compared with the activity recorded (for 250 ms) immediately before CS presentation. The integrated EMG activity recorded during the CS–US interval should be at least 2.5 times larger than the averaged activity recorded immediately before CS presentation.

### Hot-plate test

**Figure 2:**
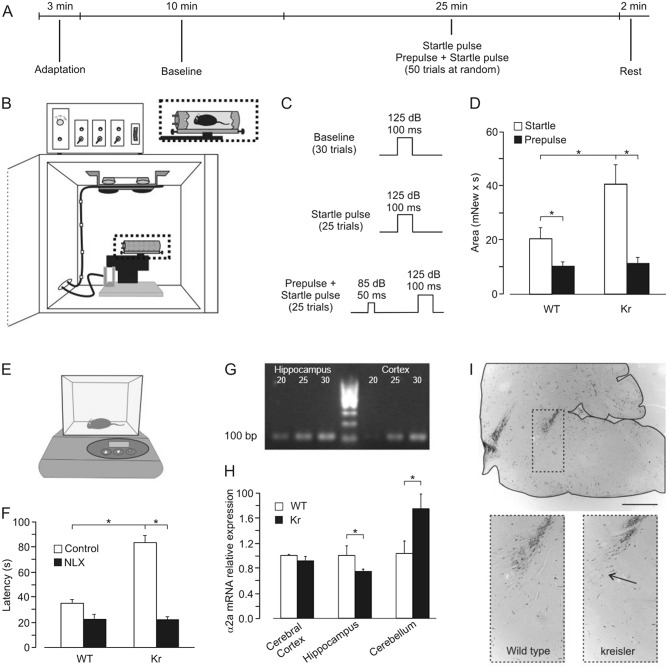
Functional characterization of kreisler mice. (**A**–**C**) A representation of the experimental design for recording the startle response and the prepulse inhibition (**A**), a diagram of the recording apparatus (**B**) and intensity (dB) and duration (ms) of the stimuli used (**C**). (**D**) Total area of the startle reflex response alone and following a prepulse presentation in wild-type (WT) and kreisler (+/kr) mice (*, *P* < 0.05; one-way ANOVA and Holm-Sidak method). (**E**) A diagram of the hot-plate device. (**F**) Effects of naloxone (NLX) administration on the latency for the behavioral response to pain during the hot-plate test in WT and +/kr mice (*, *P* < 0.001; one-way ANOVA). (**G**) DNA fragment separation in electrophoresis gel, cropped picture just to show DNA band size (100 bp). 20, 25, 30 indicate amplification cycles. (**H**) A semi-quantitative analysis of alpha2A receptor mRNA expression in prefrontal cortex, hippocampus, and cerebellum (*, *P* < 0.05; one-way ANOVA). (**I**) Immunohistochemical localization of tyrosine hydroxylase (TH) positive neurons in the locus coeruleus and in the nucleus subcoeruleus in WT and +/kr mice. Note the reduced number of TH-positive neurons in the nucleus subcoeruleus of +/kr mice. Calibration bar = 1000 µm.

Mice were screened by placing them on a hot-plate maintained at 52.2 °C^24^ (Fig. 2E). The variable measured was the ‘pain behavioral response’ defined as the time in seconds until the animal reared three times leaning against cage walls or when it jumped on the floor). Cut-off was 240 s. This experiment was carried out in eleven wild-type and ten kreisler mice.

### Acoustic startle response and prepulse inhibition

The magnitude of the startle reflex was assessed in ten wild-type and nine kreisler mice placed individually inside a startle chamber (Cibertec S.A., Madrid, Spain). The startle response was measured using a piezoelectric accelerometer controlled by a computer. The digitized signal was averaged from 25–30 recordings. Following previous descriptions from our group^23^ The experimental animal was placed in the startle chamber for an acclimation period of 3 min. Baseline responses were averaged after the presentation of 30 sounds (125 dB, 100 ms long). During prepulse inhibition trials, the same 125-dB 100-ms burst was preceded (by 250 ms) by a prepulse stimulus of 85 dB, 50 ms long. Trials including prepulse stimuli were randomly presented with normal startle stimuli, the final total being 25 of each (Fig. 2A, B and C).

### Drug administration

Naloxone (3.33 mg/kg), clonidine (Sigma-Aldrich 35 µg/kg), and idazoxan (Sigma-Aldrich 1 mg/kg), each dissolved in saline, were administered subcutaneously.

Doses used in behavioral studies were determined according to our preliminary in vivo electrophysiology tests. Drugs were given to the animals 30 min prior to the hot-plate test and 15 min to conditioning sessions.

### Immunohistochemistry

Immunohistochemistry was performed as described previously^5,8^. A total of nine control and nine kreisler mice were used in this study. Briefly, animals were deeply anaesthetized with chloral hydrate and perfused through the left ventricle with 0.1 mol/L phosphate buffer (pH 7.35) followed by freshly prepared 4% PFA in the same buffer. Brains were removed, post-fixed in the same fixative for 6–8 h, and transferred to a 30% sucrose solution in PBS for at least 3 days. Sagittal sections (35 μm) were obtained in a cryostat. They were pre-incubated in 5% normal goat serum at 4 °C for 1 h, transferred to the anti-TH antibody diluted 1:1000 in 0.1% Triton X-100 in PBS, and incubated at 4 °C for 16 h. This was followed by PBS washes and incubation in a biotinylated goat anti-rabbit antibody (Vectastain, ABC kit; Vector Labs Inc., Burlingame, CA, USA) in 0.1% Triton X-100 in PBS at 4 °C for 1 h. The tissue was then washed in PBS and incubated in an avidin–biotinylated peroxidase complex according to the manufacturer's instructions (Vectastain, ABC kit). Finally, the sections were washed in PBS and visualized with 0.05% 3,3′-diaminobenzidine in PBS and 0.009% H_2_O_2_. Staining specificity was evaluated by omission of the anti-TH antibody during the corresponding incubation, or by using a pre-immune fraction. In these cases, no labelling was observed. Neuronal counting and perceptual analysis was performed as previously indicated^8^. Briefly, percentual analysis was made by comparing pair of equivalent sections. Neuronal counting per section appear in supplementary Fig. 1.

### Alpha2A gene expression analysis by RT-PCR

Alpha2A adrenergic receptor semi-quantification was performed as described previously^25^. Briefly, total RNA from brain tissue was extracted with Tripure reagent (Roche Products, Hertfordshire, UK). A minimum of four animals per group, collected from at least two different experimental sessions, were used. Polymerase chain reaction was assayed for 25 cycles using Taq DNA polymerase. To verify exponential phase of amplification, 20 and 30 cycles were also assayed. Oligonucleotide primers 5′ ATGTTCCAGTATGACTCCACTCACC 3´ and 5′ GAAGACACCAGTAGACTCCACGACA 3′ were specific for gapdh and generated a 100 bp band; primers 5′ CAAGATCAACGACCAGAAGT 3′ and 5′ GTGCGACGCTTGGCGATCT 3′ were specific for alpha2A and generated a 100 bp band. Annealing temperature was 60 °C. Arbitrary units were calculated and compared with constitutive housekeeping gapdh expression.

### Data analysis

EMG recordings were stored directly on a computer with the help of the Signal Average Program of Cambridge Instruments (Cambridge, England) and analyzed off-line for quantification of reflex and conditioned responses^22^. Unless otherwise stated, mean values and their standard errors (SEM) are indicated. Learning curves are represented as the percentage of CRs per training session. Statistical significance was determined by one-way ANOVA for behavioral or histological analysis. The analysis of eyelid reflex and conditioned responses after drug administration was assessed by a two-way ANOVA test, comparing session-by-session and all groups. Post-hoc test used was Holm-Sidak method. The difference was considered significant for *P* < 0.05.

## Results

### Heterozygous kreisler mutation affects the acquisition of trace but not delay eyeblink classical conditioning

In a first series of experiments, we compared the learning capabilities of wild-type with those of kreisler mice using both delay and trace conditioning paradigm. As illustrated in Fig. 1A–C, both wild-type and kreisler animals presented the typical learning curve previously described in mice when we used the delay paradigm^23^. Control mice reached maximum values of the percentage of conditioned responses at the fourth session. At the end of the conditioning protocol (eight sessions), the two groups of animals reached similar values (around 70% of conditioned responses). Nevertheless, the acquisition rate was significantly slower in kreisler mice as compared with their littermate controls. During the fourth and fifth sessions, kreisler mice presented a lower percentage of conditioned responses than the control group (Fig. 1C). However, when we used the trace paradigm, kreisler animals were unable to acquire conditioned responses across training, presenting no significantly different values from those collected during habituation sessions (Fig. 1D–F). In contrast, wild-type mice evolved normally during the trace conditioning task, reaching values around 60%, as was previously described when we applied the same paradigm^22^.

### A pharmacological study of the differences in the startle response test between wild-type and kreisler mice

We tested the startle response and prepulse inhibition in both groups of animals (Fig. 2A–D). The latency of the startle response was similar for the two groups of animals (23.00 ± 0.86 ms for wild-type and 24.64 ± 0.80 ms for kreisler; F_(1,18)_ = 1.97, *P* = 0.18; not shown). However, as shown in Fig. 2D, kreisler mice presented a higher intensity of the total area of the startle response compared with the wild-type group (20.48 ± 3.91 mNew x s for wild-type vs 40.39 ± 7.43 mNew x s for kreisler; F_(1,18)_ = 5.97, *P* = 0.025), indicating an evident hyper-reactivity to the sudden presentation of the strong sound stimulus. In contrast, kreisler mice presented no significant differences with control animals in the startle response in the presence of prepulse inhibition (10.03 ± 1.72 for wild-type against 11.20 ± 2.28 mNew x s for kreisler; F_(1,18)_ = 0.17, *P* = 0.68).

### Differences in the behavioral response to pain during the hot-plate test between wild-type and kreisler mice

During the hot-plate test, the kreisler group presented higher latencies for the behavioral response to pain than the wild-type group (35.29 ± 2.69 s for wild-type vs. 83.35 ± 6.18 s for kreisler mice; F_(1,20)_ = 54.37, *P* < 0.001; Fig. 2E, F). As it has been reported that kreisler mice present an overactivation of the opioid system during the first postnatal days (6), we tested the same behavioral response to pain under the pharmacological inhibition of opioid receptor with naloxone. Under these conditions, kreisler latencies appeared normal (22.47 ± 4.09 s in controls vs. 22.14 ± 2.31 s in kreisler mice; F_(1,20)_ = 0.95, *P* = 4.38), showing a significant decrease compared with control values (F_(1,19)_ = 86.14, *P* < 0.001), indicating that the opioid system is still overactivated in adult kreisler mice.

### Quantification of alpha2A receptor with RT-PCR

As the startle reflex and its posterior inhibition when a prepulse is presented is regulated by the noradrenergic system, we decided to check alpha2A receptor levels in different regions of the mice brains. It is well known that noradrenergic axons project to and reach many rostral regions. In accordance, we dissected out tissue from three representative brain areas (prefrontal cortex, hippocampus, and cerebellum) and isolated mRNA expressed by them. By semi-quantitative RT-PCR we compared the expression of alpha2A receptor mRNA in kreisler samples with those collected from wild-type mice. As shown in Fig. 2G, H, there were no differences in the relative expression of that receptor at the level of the prefrontal cortex (1 ± 0.10 in controls vs. 0.95 ± 0.04 in kreisler mice; F_(1,7)_ = 0.18, *P* = 0.69). We obtained a significant decrease in the relative expression at the level of the hippocampus (1 ± 0.09 in wild-type vs. 0.74 ± 0.05 in kreisler mice; F_(1,7)_ = 6.43, *P* < 0.05) and an increase at the level of the cerebellum in kreisler mice (1 ± 0.14 in wild-type vs. 1.77 ± 0.19 in kreisler mice; F_(1,7)_ = 10.83, *P* < 0.05). Although this technique is not fully precise, it is telling us that the expression of alpha2A receptors is modified in areas that are very important in tasks related to learning processes, such as the hippocampus and the cerebellum. In any case, we must consider that this is only mRNA levels, not receptor levels for which autoradiography, or other techniques, should be used.

### Immunohistochemistry for tyrosine hydroxylase

Even when exhibiting a normal appearance, heterozygous kreisler mice have been shown to suffer a modified embryonic gene expression pattern, located mainly in the rostral hindbrain^13^. Data collected from prepulse inhibition of the startle reflex, as well as quantification of cortical and hippocampal adrenergic receptors, pointed to the locus coeruleus/subcoeruleus region. Accordingly, we located noradrenergic neurons in these areas by using immunohistochemistry for tyrosine hydroxylase (TH). As shown in Fig. 2I, heterozygous kreisler mice present a normal size of the locus coeruleus but have an evident reduction in the number of neurons at the level of the subcoeruleus region (100 ± 13.69% in wild-type vs. 55.12 ± 8.69% in kreisler mice; F_(1,17)_ = 7.65, *P* < 0.05).

### Effects of agonist and antagonist co-administration of drugs on corneal reflex responses

**Figure 3:**
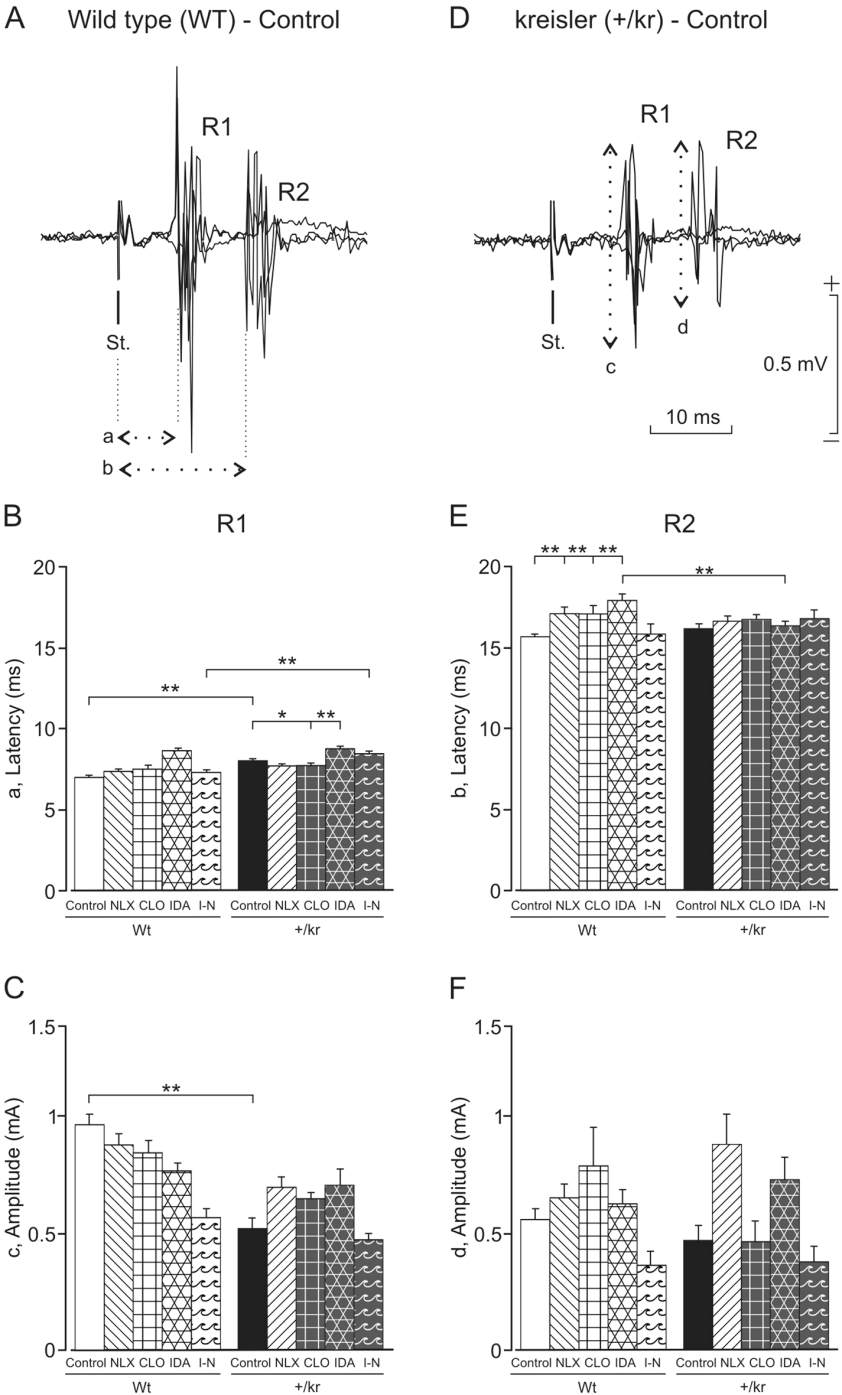
Reflexively evoked blinks in wild-type and kreisler mice. (**A**, **D**) Three superimposed EMG traces recorded from the orbicularis oculi muscle in a wild-type control (WT, **A**) and in a kreisler control (+/kr, **D**) animal following a single electrical stimulation (50 µs, 2 × Threshold) of the supraorbital nerve. Note the presence of the characteristic R1 and R2 components of the blink reflex. The latencies of R1 (a) and R2 (b) components, as well as their respective amplitudes (c and d), were quantified as indicated by the double-arrowed, dotted lines. (**B**, **C**, **E**, **F**) Mean values collected for latency (**B** for R1, E for R2) and amplitude (**C** for R1, F for R2) of both R1 and R2 components of electrically evoked blinks in WT and +/kr mice before (controls) and after the administration of naloxone (NLX), clonidine (CLO), idazoxan (IDA), and idazoxan plus naloxone (I-N). Significant differences were observed between groups for the latency of R1 and R2 and the amplitude of the R1 component (F_(3,4303)_ = 12.56; *, *P* < 0.05; **, *P* < 0.001; two-way ANOVA and Holm-Sidak method).

In order to test the functionality of neural circuits involved in eyelid reflex responses in wild-type and in kreisler mice, we electrically stimulated the supraorbital nerve and recorded electromyographic (EMG) responses evoked in the orbicularis oculi muscle (Fig. 3A, D). The corneal reflex presents an early (R1) and a late (R2) EMG response of the orbicularis oculi muscle previously described^23,26^. Kreisler mice generate the two (R1 and R2) components of the eyelid reflex response, but the R1 response appeared significantly later (8.04 ± 0.11 ms; *P* ˂ 0.001) and was smaller (0.52 ± 0.04 mV; *P* ˂ 0.05) than in wild-type mice (6.99 ± 0.07 ms and 0.96 ± 0.04 mV; Fig. 3B, E).

The administration of clonidine and idazoxan altered the latency of appearance of R1 in wild-type mice (clonidine: 7.49 ± 0.13 ms; idazoxan: 8.68 ± 0.15 ms; *P* ˂ 0.001) and R2 (clonidine: 17.07 ± 0.52 ms; idazoxan: 18.01 ± 0.22 ms; *P* ˂ 0.001) components of the corneal reflex. In contrast, in kreisler mice the latency of only the R1 component was affected (clonidine: 7.71 ± 0.09 ms, *P* ˂ 0.05; idazoxan: 8.72 ± 0.13 ms, *P* ˂ 0.001).

A two-way ANOVA F-test (F_(3,4303)_ = 12.57; *, *P* < 0.05; **, *P* < 0.001) indicated that only the latency of R2 in wild-type mice was modified by the administration of naloxone (wild-type control: 15.75 ± 0.19 ms; wild-type naloxone: 17.12 ± 0.32 ms; *P* ˂ 0.001; Fig. 3E). The differences found in the latency of R1 between wild-type and kreisler mice described above disappeared with the administration of naloxone, clonidine, and idazoxan, but not when idazoxan and naloxone were administrated together (*P* ˂ 0.001). Idazoxan administration increased the latency of R2 in wild-type mice (wild-type control: 15.75 ± 0.19 ms; wild-type plus idazoxan: 18.01 ± 0.22 ms; *P* ˂ 0.001), leading to significant differences between kreisler and wild-type mice (R2 latency in kreisler plus idazoxan: 16.43 ± 0.2 ms; *P* ˂ 0.001). The amplitudes of R1 and R2 did not change with the administration of any drug (Fig. 3C, F).

### Effect of agonist and antagonist co-administration of drugs on the acquisition of classically conditioned eyelid responses

**Figure 4:**
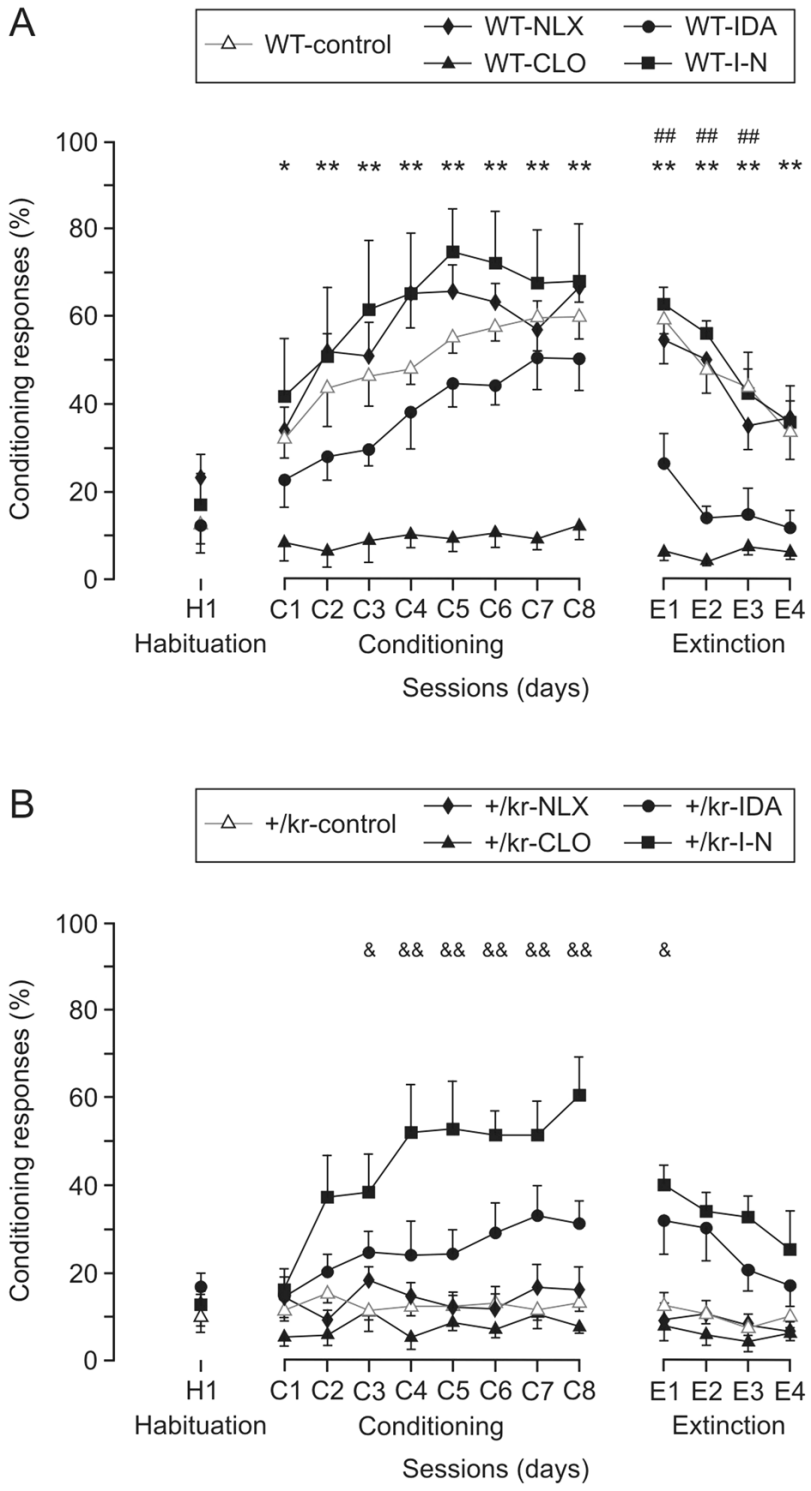
Learning curves for wild-type (**A**) and kreisler (**B**) mice during trace conditioning paradigm. Evolution of the percentage (%) of conditioned responses (CRs) across sessions in the two (WT and +/kr) groups of mice, without (controls) and with the administration of naloxone (NLX), clonidine (CLO), idazoxan (IDA), and idazoxan plus naloxone (I-N). Data collected from the 2nd habituation session (H2), C1–C8 conditioning sessions, and E1–E4 extinction sessions are illustrated. The percentage (± SEM) of CRs is indicated. Gray lines indicate control values for wild-type (WT) and kreisler (+/kr) mice. Significant differences between wild-type control (WY-control) and wild-type plus clonidine mice (WT-CLO, *), wild-type control (WT-control) and wild-type plus idazoxan mice (WT-IDA, #), and kreisler control (+/kr-control) and kreisler plus idazoxan and naloxone groups (+/kr-I-N, &), were statistically different (F_(12,715)_ = 2.148; *, ^&^, *P* < 0.05; **^, &&, ##^, *P* < 0.001; two-way ANOVA and Holm-Sidak method). Only the significant differences compared with the control groups are represented in the figure.

As illustrated in Fig. 1F, when a trace conditioning paradigm was used, control wild-type mice presented a performance of 60.1 ± 5.19% of conditioned responses at the end of the 8th session (see also the gray line in Fig. 4A). An experimental treatment with naloxone had no effect on the ability of those mice in acquiring similar percentages of conditioned responses. Wild-type mice treated with idazoxan seemed to obtain lower percentages of conditioned responses (60.1 ± 5.19% vs. 50.25 ± 7.19%) by the eighth conditioning session, although differences were not significant. However, during the first extinction sessions the decrease in the percentage of conditioned responses was significantly faster than in control mice (59.64 ± 3.87% in WT control vs 26.58 ± 6.87% in WT plus idazoxan). Surprisingly, wild-type mice treated with clonidine were unable to acquire conditioned responses. Combined treatment with naloxone and idazoxan produced slightly higher non-significant conditioned responses than in controls (12.45 ± 3.46% in WT plus clonidine during the eight conditioning sessions).

We have already described that kreisler mice are unable to acquire conditioned responses when the trace paradigm is used (Fig. 1F) and that they also present some significant deficits in both the opioid and adrenergic systems (Fig. 2). A pharmacological study appeared a logical approach in order to inquire if those deficits could also be involved in the acquisition of associative learning tasks such as the hippocampal-dependent classical eyeblink conditioning using a trace paradigm^27^. Kreisler mice treated with clonidine or naloxone never increased the rate of conditioned eyeblink responses obtained during habituation sessions (8.28 ± 1.54% in +/Kr plus clonidine and 16.95 ± 4.51% in +/kr plus naloxone vs 13.66 ± 2.01% in +/Kr control, during the eight conditioning sessions; Fig. 4B). A treatment with idazoxan seemed to increase the percentage of conditioned responses in kreisler mice, moving from 17.05 ± 2.98% during the second habituation session (H2 in Fig. 4B) to 33.30 ± 6.78% during the seventh conditioning session (C7 in Fig. 4B), although differences were not significantly different. In contrast, a significant increase in the acquisition of conditioned eyelid responses was obtained when we treated kreisler mice with both naloxone and idazoxan: the percentage of conditioned responses increased significantly to 60.69 ± 8.70 [(F_(12,715)_ = 2.148; *, ^&^, *P* < 0.05; **^, &&, ##^, *P* < 0.001; two-way ANOVA]. These results suggest that the proposed malfunction of both opioid and adrenergic systems was affecting the normal acquisition of conditioned responses in this mouse model and that we were able to restore this associative learning capability by using the indicated pharmacological approach.

## Discussion

In the present work, we have analyzed behavioral and cognitive processes in adult heterozygous kreisler mutant mice. During embryonic development, they suffer ectopic gene expression in the rostral hindbrain. They are born with a normal appearance, and they can reach adult ages.

An overall view of the results demonstrates the role of the noradrenergic system in the acquisition of conditioned motor responses. In our case, we describe how clonidine prevents the appearance of eyelid conditioned responses in wild-type mice. This is one of the most interesting results obtained from this work. Adult kreisler mice presented the typical learning curve previously described in mice when we used the delay paradigm. However, they were unable of acquiring conditioned responses across training when the trace paradigm was used. Anatomical and molecular characterization pointed to A5 neurons and noradrenergic functional projections to the hippocampus. Pharmacological simultaneous treatment with idazoxan and naloxone increased the percentage of conditioned responses to wild-type levels, suggesting that both systems are affected in kreisler mice, and even that there is a synergy between the two receptor systems in the control of the acquisition of conditioned responses.

Firstly, using two different paradigms we have addressed the study of the ability of heterozygous kreisler mice to acquire eyelid conditioned responses. The reason for using this animal model and the conditioning of the eyelid response is that in the different characterizations carried out on this mouse model there are characteristics that differentiate it from the homozygous mutant and from the wild type. Genetic^13^, morphological^6^, and functional^6,21^ alterations have been detailed in the heterozygous kreisler mutant that justify the choice of this model to carry out the study.

Studies performed in mice affected in different regions of the brainstem have demonstrated that an antiapneic system located in the rostral pons controls the postnatal respiratory frequency^28^. It is well known that this system is influenced by the action of the coeruleus and subcoeruleus adrenergic systems. Both systems have a significant role in the neonatal respiratory maturation, and A5 neurons are known to slow down such activity^14,29^. At birth, heterozygous kreisler mice present increased respiratory frequencies and a hyperactivated opioid system^6^. We now demonstrate in kreisler mice a 50% decrease in the number of A5 neurons in the brainstem, which could be responsible for that increased respiratory frequency. This is promising because mutant mice exhibiting a higher magnitude of subcoeruleus nucleus, for example Krox20 mutant mice, presented apneas and low respiratory frequency^4^. Working with ponto-bulbar in vitro preparations in these heterozygous kreisler mice, problems have also been demonstrated related to the response to hypoxia. Some of the areas implicated in this response involved the A5 group^21^. In any case, it is difficult to explain how this A5 reduction appears during development, but we can consider the possibility of the mutation affecting specific neurons inside the nucleus, as it has been demonstrated that different populations coexist in such nucleus, according to the expression of molecular markers^30^. As early brainstem postnatal deficiencies may be maintained in younger/adolescent mice^5^, and heterozygous kreisler mice survived and presented no anatomical or behavioral deficits, we considered the possibility of having the same malfunction of adrenergic and opioid system in kreisler mice older than 3 months. We considered that the brainstem regions affected in kreisler mice, and their projections, could interfere with the execution of eyelid reflex responses and, consequently, affect motor control and learning.

Eyelid classical conditioning is a well-established model to study the cellular mechanisms underlying associative learning and memory^31,32^. With this paradigm, we can evoke a reflex eyeblink in the intact animal in response to a sound when it is coupled to an airpuff presented to the cornea. We had also successfully assayed the airpuff/AIRPUFF paradigm^27^, as well as a modification of this technique combining, in mice, two electrical stimuli^22^. Brainstem, hippocampus, and cerebellum have been proposed as essential components of the neuronal circuits involved in those responses in adults^33–35^, which we have also demonstrated recently^23,36^.

Of course, before trying conditioning, we tested in kreisler mutants the ability to generate eyeblink reflex responses. They are present, although both the latency and the amplitude of R1 are reduced. We were able to observe the appearance of the two components of the reflex response, R1 and R2. Thus, the neuronal circuit that generates this response remains intact. We will discuss this later. As we have mentioned, classical conditioning depends on the experimental paradigm used. Delay conditioning depends on the activity of the cerebellum^23,36,37^. When this paradigm was used, kreisler mice presented an almost normal acquisition curve, suggesting that there is no functional alteration in acquisition. When using the trace paradigm in the classical eyelid conditioning, the acquisition of conditioned responses depends on the activity of the hippocampus^22,38^. Kreisler mice are impaired in this type of learning, indicating that this structure might be functionally affected. Consequently, we performed some behavioral, molecular, and anatomical studies.

Firstly, we have demonstrated that the opioid system is still overactivated in adult kreisler mice. We had previously demonstrated the same state in newborn mice^6^. Latencies in the response to hot-plate are high in kreisler mice compared with those in wild-type mice, and they become normal after naloxone administration. So, the hyperactivated opioid system is producing this analgesic effect. Secondly, we have tested a possible malfunction of the noradrenergic system. This has been demonstrated to participate in the startle response and in the prepulse inhibition, related to rostral projections from the brainstem to the prefrontal cortex^18,19^. Kreisler mice present an increased startle response, like that of mice treated with alpha2 agonist^18^. The intensity of this response is inhibited following a prepulse. This suggests that the noradrenergic system is affected in kreisler mice, and points to the participation of alpha2 receptors, as has also been shown previously in humans^39^. Modification in this system is also demonstrated by mRNA expression quantification of alpha2a receptors in different cerebral areas. Our biochemical analysis demonstrates that our mutant mice present a significant reduction in the expression of alpha2a receptor in the hippocampus, thus suggesting that the impairment in the acquisition of conditioned responses using the trace paradigm could be explained by such receptor deficit. Interestingly, expression of these receptors in the cerebellum is increased, and seems not to affect the acquisition of conditioned responses using the delay paradigm. As it is well known that noradrenaline participates in this paradigm^40^, we can assume that the release of noradrenaline in kreisler cerebellar structures is normal or not affecting its function. Furthermore, the noradrenergic system seems to be structurally affected, as anatomical analyses demonstrated a reduction in the number of A5 neurons in the brainstem. This is very interesting, as lesions located at the level of the dorsal A5 have been demonstrated to impair consolidation of emotional memory, such as that of context-associated fear conditioning^41^. As the acquisition of conditioned responses using the trace paradigm is impaired in kreisler mice, we focused on this paradigm and on the involvement of the hippocampus in order to test the effect of different drugs.

In our investigation, kreisler mice presented the reflex response to the palpebral stimulus (R1 and R2), but with a delay compared with the control group, especially in the R1 component. The amplitude of the signal, likewise, appears diminished in this mutant. The presence of deficits in motor-sensory integration at the level of the brainstem is revealed in kreisler mice, involving the noradrenergic system, in agreement with data presented previously in mice treated with clonidine^42^. In fact, numerous studies show that the noradrenergic system (specifically the α2 receptors) modulates the latency and amplitude of the R1 and R2 components^43,44^. However, it is also true that this is dose dependent, as systemic administration of very low doses of alpha 2a agonist or antagonist had no significant effects on blink reflex parameters^45^. In cases of anxiety, in which the central noradrenergic system is also involved, reflex responses have also been shown to be delayed^46^. Very interesting is the case of clonidine administration in humans, in which, even at a very low dose of 0.5 µg/kg, the limitation in the release of norepinephrine reduces the amplitude of the R1 component^43^. We have now demonstrated this in wild-type animals, and this leads us to suspect again that kreisler mice function with limited release of noradrenaline.

In our study, the differences in the amplitude of R1 between the wild-type group and the kreisler group disappear with the isolated administration of the drugs, but not, surprisingly, when idazoxan and naloxone are administered together. Regarding the R2 component, considered to be a nociceptive response, it has been shown that it is modulated by the release of endogenous opiates and is affected by the administration of naloxone^47^. This may explain why we obtained an increase in the latency of this component when we administered it to wild-type mice. It should be noted that in our case the amplitudes of R1 and R2 did not change with the administration of any drug.

After showing that both eyelid reflex (R1 and R2) components are present, we can discuss the main problem in kreisler mice. Considering anatomical and functional deficiencies affecting the pontine noradrenergic system, we expected to find deficiencies in cognitive function in adult kreisler mice, as both noradrenergic and opioid systems have been demonstrated to participate in the acquisition of conditioned responses^48–50^.

Looking at the role of the different systems in the control of learning processes, there is evidence of the participation of the opioid system, as well as the effect of treatments involving the adrenergic system. Regarding the former, it has been described how the administration of opioid agonists affects, for example, spatial learning^51^. They also affect the acquisition of classically conditioned responses in rabbits, involving kappa or mu receptors. This effect is antagonized by naloxone^52,53^. In our case, it seems logical that the hyperactivity of the opioid system in kreisler mice, present in adults and corroborated with the nociceptive test, could affect the acquisition of conditioned eyelid responses, although treatment with naloxone, which normalizes the behavioral response to pain, does not reverse such deficiency. In our case, naloxone alone did not affect conditioned responses in wild-type mice, as has been demonstrated previously in rabbits^53^. Results obtained with naloxone are sometimes controversial, as in many cases its action depends on doses used, administration time, or conditioning characteristics^50,54^. If we exclude those studies involving aversive or painful stimuli and the involvement of the prefrontal cortex, we can focus on hippocampal opioid receptors in learning and memory. Their role remains presenting complexity. For example, hippocampal mu opioid receptors can have a positive role in learning^55^ or produce an impairment of spatial ability^56^. More abundant are the articles investigating the participation of the noradrenergic system in learning processes. It is well documented that noradrenaline when injected into the amygdala, hippocampus, or entorhinal cortex enhances memory formation^57,58^. We must consider once more the complexity present in this system, with functional differences arising from cell population heterogeneity in the locus coeruleus^59^, which can explain differences observed in emotional associative learning, contextual learning, or cognitive flexibility, following adrenergic activation^60–62^. Adrenergic alpha2 receptor activation both facilitates^63^ and impairs learning^64^, depending on the learning paradigm or administration. One of the most interesting results of our study is that obtained following treatment with clonidine. It is noteworthy that this treatment impaired associative learning in the same way as has been described in other learning approaches, such as fear conditioning^65^. In a recent work with adolescent mice, we have demonstrated, using a novel object recognition task, that clonidine treatment affects acquisition of short-term memories^42^. The dose used has been reported useful in the activation of presynaptic alpha2a receptors and sufficient to decrease the spontaneous firing rate of locus coeruleus neurons and the release of norepinephrine in cortical areas^66^. This result suggests that the kreisler mouse, in which we show that the noradrenergic system is functionally affected, may be behaving like the control mouse when it works under the effect of clonidine. This is why, with the intention of increasing the release of norepinephrine in these mice, we used other drugs such as idazoxan, which increases the NA release even at low doses^67^ and facilitates memory^68^. Similar results had previously been obtained with other alpha2 antagonists^69^.

Although many studies have focused on the locus coeruleus when talking about the noradrenergic system, the role of the subcoeruleus nucleus in learning processes has also been investigated. It has been demonstrated that lesions in the dorsal subcoeruleus nucleus impair consolidation of context-associated fear conditioning memories^41^. It is interesting to note that kreisler mice have this nucleus affected, showing a reduction of almost 50% in the number of neurons, and that we had previously shown an increase in respiratory rate in newborn mice^6^. Although it is still unresolved whether A5 acts directly with its rostral projections or through its projections to the locus coeruleus, our study proposes that the intact A5 is necessary for the acquisition of conditioned responses. A neuroprotective role of noradrenaline has recently proposed in cases of animal models with impaired noradrenergic system. Interestingly, in some cases functional noradrenergic degeneration occurs without Th positive cell loss^70,71^. For us is important to consider that projections to Hippocampus could be unaltered and we could still observe malfunction of the noradrenergic system. It must be borne in mind that opioid and adrenergic systems are functionally related. It is well accepted that opioids can inhibit neurons in the locus coeruleus^72,73^. Thus, for our study, it is very important to highlight those cases in which the joint participation of the two neurotransmitter systems has been investigated. Both synergy and allosteric effect have been proposed when drugs for the two systems are co-administered, mainly in studies involving analgesia^74–77^. In many cases, the administration of opioid agonists and adrenergic antagonists, which affect the release of norepinephrine, affect learning. As it has even been proposed that opioid and adrenergic receptors may form heterodimeric receptors^78^, this could explain why ineffective doses when applied alone proved effective when administered together. Highlighting the novelty of our study, the opposite effect had not been investigated, with joint treatments aimed at increasing the release of noradrenaline. In our study, the low dose of idazoxan used slightly increased the acquisition of conditioned responses (the trend increased, although not significantly). As we have mentioned, administration of naloxone alone had no effect, but the joint administration of the two substances achieved a percentage of conditioned responses like that of the control group. This conclusion is promising, since the joint administration of idazoxan and naloxone could be effective in cases of cognitive deficiencies in which a functional alteration of the noradrenergic system is suspected.

## Supplementary Information

Supplementary Information 1.

Supplementary Information 2.

Supplementary Information 3.

Supplementary Information 4.

## Data Availability

Derived data supporting the findings of this study, word embedding and text mining data, are available to the corresponding authors upon request.
