# Supplementary material for: Noradrenergic projections regulate the acquisition of classically conditioned eyelid responses in wild-type and are impaired in kreisler mice
Source: Sci Rep. 2023 Jul 15;13:11458. doi: 10.1038/s41598-023-38278-4 (PMC10349844; doi:10.1038/s41598-023-38278-4)
Supplement: Supplementary file 1 — Supplementary Information 1. [file 41598_2023_38278_MOESM1_ESM.docx]

**Supplementary Figure 1.** Immunohistochemical localization of tyrosine hydroxylase in the nucleus subcoeruleus in Wild type and Kreisler mice. (A). Serial sections showing the reduced number of TH-positive neurons in the nucleus subcoeruleus of Kreisler mice. (B). Quantification of TH-positive neurons per section. (C). Mean value of neurons per section are 31.44 ± 4.3 for Wild type, and 17.33 ± 2.73 for Kreisler mice (F_(1,17)_ = 7.657; *, P < 0.05, one-way ANOVA). Calibration bar= 250 µm.
