# Supplementary material for: Noradrenergic projections regulate the acquisition of classically conditioned eyelid responses in wild-type and are impaired in kreisler mice
Source: Sci Rep. 2023 Jul 15;13:11458. doi: 10.1038/s41598-023-38278-4 (PMC10349844; doi:10.1038/s41598-023-38278-4)
Supplement: Supplementary file 2 — Supplementary Information 2. [file 41598_2023_38278_MOESM2_ESM.docx]

**Supplementary Figure 2.** DNA fragment separation in electrophoresis gel. Gel edges are not visible in the picture, but empty wells are visible. All lines correspond to DNA amplificated for alpha2A gene except one corresponding to DNA size marker. DNA loaded correspond to mRNA originally isolated from cerebellum, hippocampus and cerebral cortex from three animals, two WT (WT-03 and WT-04) and one Kr (Kr-02). 100 bp indicate amplificated DNA band size. 20, 25, 30 indicate amplification cycles.
