# Supplementary figures and images for: Noradrenergic projections regulate the acquisition of classically conditioned eyelid responses in wild-type and are impaired in kreisler mice
Source: Sci Rep. 2023 Jul 15;13:11458. doi: 10.1038/s41598-023-38278-4 (PMC10349844; doi:10.1038/s41598-023-38278-4)

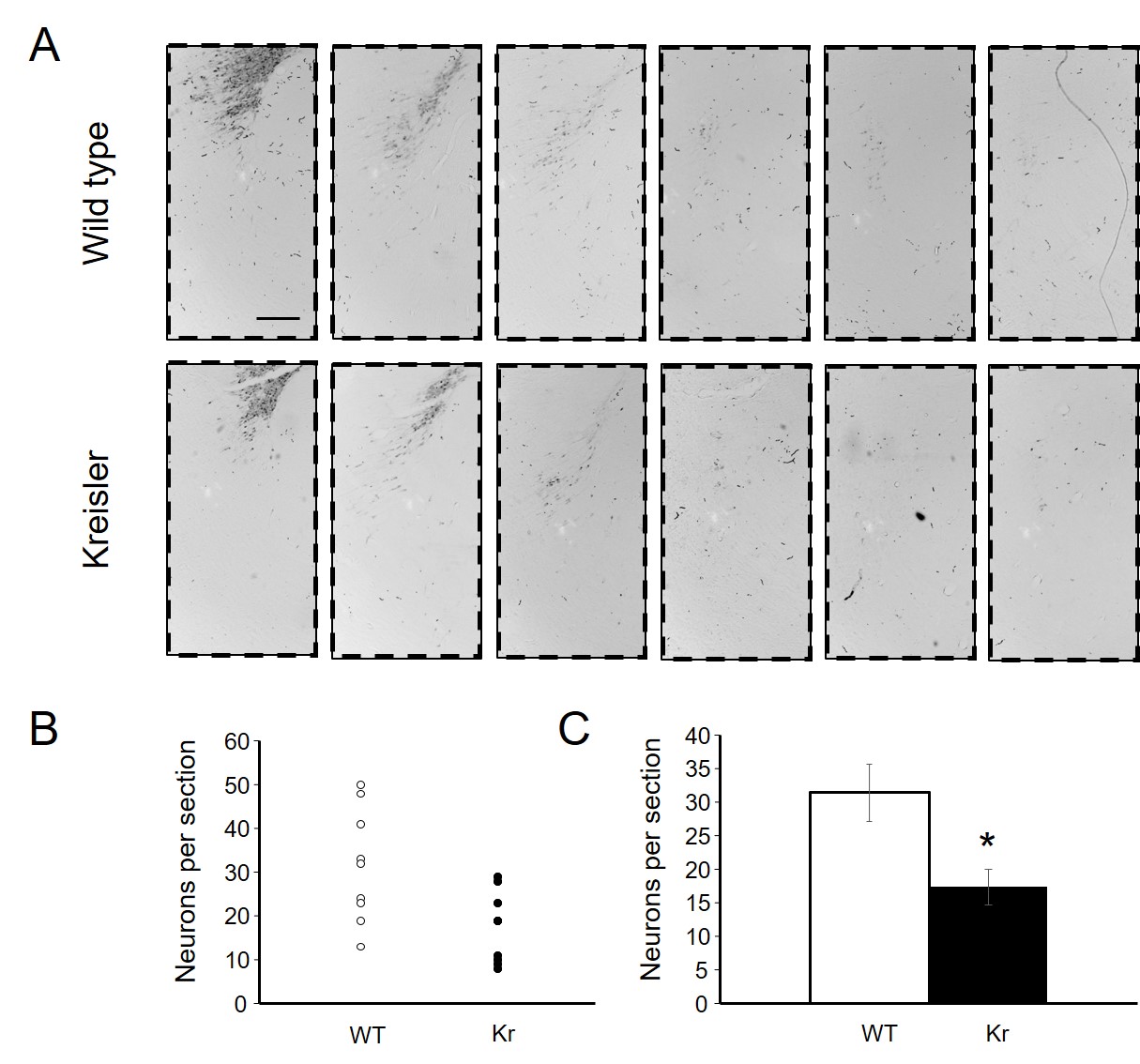

Supplement: Supplementary file 3 — Supplementary Information 3. [file 41598_2023_38278_MOESM3_ESM.jpg]

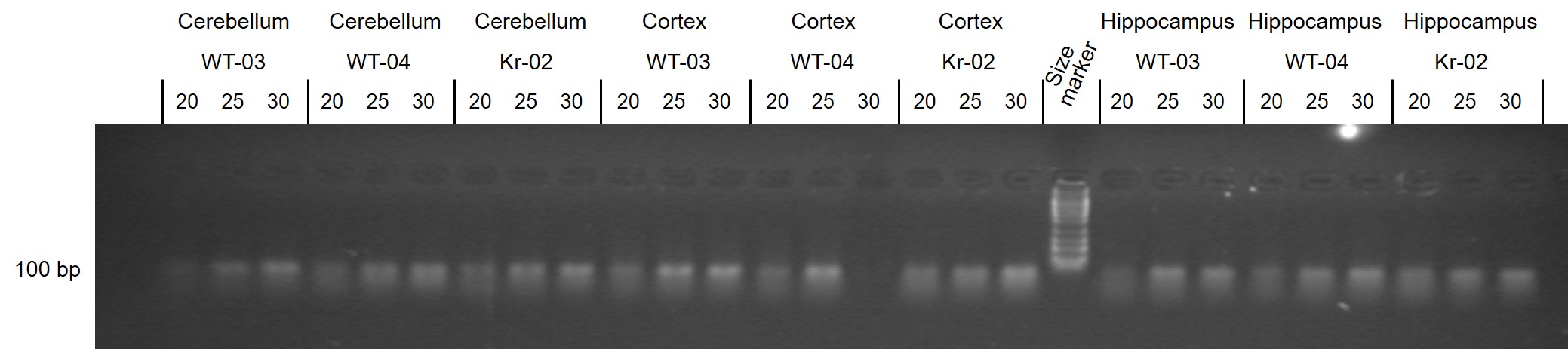

Supplement: Supplementary file 4 — Supplementary Information 4. [file 41598_2023_38278_MOESM4_ESM.jpg]
